# An Opportunity for Pharmacists to Help Improve Coordination and Continuity of Patient Health Care

**DOI:** 10.3390/pharmacy6030078

**Published:** 2018-08-01

**Authors:** Jon C. Schommer, Lawrence M. Brown, Ryan (Alyssa) Bortz, Alina Cernasev, Basma T. Gomaa, Keri D. Hager, Lisa Hillman, Olihe Okoro, Serguei V. S. Pakhomov, Paul L. Ranelli

**Affiliations:** 1 College of Pharmacy, University of Minnesota, 308 Harvard Street, SE, Minneapolis, MN 55455, USA; bortz011@umn.edu (R.A.B.); cerna004@umn.edu (A.C.); gomaa002@umn.edu (B.T.G.); hill0667@umn.edu (L.H.); pakh0002@umn.edu (S.V.S.P.); 2 School of Pharmacy, Chapman University, Rinker Health Science Campus 205, 9401 Jeronimo Rd #116, Irvine, CA 92618, USA; lbbrown@chapman.edu; 3 College of Pharmacy Duluth, University of Minnesota, 1110 Kirby Drive, Duluth, MN 55812-3003, USA; khager@umn.edu (K.D.H.); ookoro@d.umn.edu (O.O.); pranelli@d.umn.edu (P.L.R.)

**Keywords:** pharmacist, workforce, demand, supply

## Abstract

Pharmacist workforce researchers are predicting a potential surplus of pharmacists in the United States that might result in pharmacists being available for engagement in new roles. The objective for this study was to describe consumer opinions regarding medication use, the health care system, and pharmacists to help identify new roles for pharmacists from the consumer perspective. Data were obtained from the 2015 and 2016 National Consumer Surveys on the Medication Experience and Pharmacist Roles. Out of the representative sample of 36,673 respondents living in the United States, 80% (29,426) submitted written comments at the end of the survey. Of these, 2178 were specifically about medicines, pharmacists or health and were relevant and usable for this study. Thematic analysis, content analysis, and computer-based text mining were used for identifying themes and coding comments. The findings showed that 66% of the comments about medication use and 82% about the health care system were negative. Regarding pharmacists, 73% of the comments were positive with many commenting about the value of the pharmacist for overcoming fears and for filling current gaps in their healthcare. We propose that these comments might be signals that pharmacists could help improve coordination and continuity for peoples’ healthcare and could help guide the development of new service offerings.

## 1. Introduction

Pharmacist workforce researchers are predicting a potential surplus of pharmacists in the United States within the next 10 years [1,2,3] that might result in pharmacists being available for engagement in new roles, especially if pharmacists can provide new products and services that are not yet offered [4,5]. For there to be new roles for pharmacists, not only does there need to be untapped capacity [4,5] there must also be consumers’ willingness to accept new products and services from pharmacists [6,7,8,9,10,11].

There is evidence of pharmacist capacity for serving new patient care roles [5]. Consumers typically have a favorable overall perception of pharmacists [6,7,8,9], are in favor of the development of extended professional services [6,7,8], and would be willing to accept pharmacist-provided medication management services [9]. However, many pharmacists have been dedicated to professional services associated with medication dispensing and have been unavailable for providing services directly to consumers beyond their medication dispensing responsibilities [8,9,10,11].

Arora and colleagues [12] measured both *unmet demand* and *latent demand* for pharmacists at the state level during 2011–2012. They operationalized *unmet demand* as the “number of vacant budgeted positions in pharmacies” [12]. That is, these were funded and available positions for pharmacists at a point in time. Their findings showed that 1.5% of full time equivalent pharmacist positions were vacant and that 5.1% of the 1175 pharmacies in the state had at least one pharmacist vacancy. They operationalized *latent demand* as the “number of additional unbudgeted full-time equivalent (FTE) pharmacist positions the pharmacy needed to meet current demand for pharmacy goods and services” [12]. Their findings showed that 28.2% of the pharmacies had the presence of latent demand for pharmacists that totaled 393 additional FTEs (9.5% of the current pharmacist workforce in that state).

Findings reported by Arora et al. [12] suggest that there is a balance between the supply and demand in the United States for pharmacists but there is also a latent demand for pharmacists who could be hired to meet current needs if pharmacists and resources were available. With the predicted surplus of pharmacists [1,2,3], it appears that there will be a supply of pharmacists to meet the latent demand. However, will hiring organizations devote the resources needed to hire more pharmacists? To help make decisions about resource allocation, an assessment of *latent markets* can help an organization decide what new products and services will be most in demand in the future. Latent markets are potential markets (http://www.businessdictionary.com) that can be identified for a product or service that is not yet offered. To help gain insights about what these latent markets might be, the objective for this study was to describe consumer opinions regarding medication use, the health care system, and pharmacists—given in their own words—to help identify new roles for pharmacists that could be developed into new product and service offerings.

## 2. Materials and Methods

### 2.1. Sample and Data Collection

Data were obtained from the 2015 and 2016 National Consumer Surveys on the Medication Experience and Pharmacist Roles (Schommer and Brown, Principal Investigators). Data were collected from adults residing in the United States via on-line, self-administered surveys coordinated by Qualtrics Panels between April 28 and June 22, 2015 (*n* = 26,173) and between March 14 and 30, 2016 (*n* = 10,500).

Both surveys asked questions about respondents’ medication experiences in the main part of the surveys. The surveys differed slightly in how they finished, however. The 2015 survey finished with questions about respondent demographics and opinions regarding advertising of medications. The 2016 survey finished with questions about health-risk behaviors and respondent demographics. Then, at the very end of each survey, the following ending question was asked: “Thank you for completing this survey. If you have any comments about medicines, pharmacists, or health, please write them in the space provided.” Written responses to that ending question served as the data source for the findings presented in this report.

Of the 36,673 respondents, 80% (29,426) submitted written comments at the end of the survey. Most of the comments were about the survey (such as, “enjoyed the survey” or “thanks for this opportunity”). However, 2178 of the comments were specifically about medicines, pharmacists or health and were relevant and usable for this study.

### 2.2. Thematic Analysis

Thematic analysis of the usable and relevant written comments was conducted in order to identify overall categories that could be used for subsequent content analysis [13,14,15]. The comments were read several times by six investigators (AC, BG, KH, LH, OO, JS) independently and the main themes were extracted [15]. Comments referring to a particular theme were grouped and further explored and compared with initial key ideas [15,16]. Once the initial analysis was carried out, the interpretations were discussed among the six researchers who conducted the thematic analysis and one additional colleague who was part of the project team (PR).

### 2.3. Content Analysis

After thematic interpretations were discussed, the additional colleague who was part of the project team (PR) developed an initial list of coding categories for subsequent content analysis of the comments. This list was reviewed, modified, and operationalized by four researchers (AC, BG, LH, JS) who met in person for this purpose. All investigators agreed upon major themes [17] and operational definitions of each are presented in Table 1.

Four researchers (AC, BG, LH, JS) were trained to conduct coding for a relatively small number of comments to assess inter-judge reliability. The researchers were trained on the rules and procedures for coding, and they independently scored each comment. Inter-judge reliabilities were then calculated for 136 coding decisions by using the Perrault and Leigh reliability index (I), as follows: I = {[(F/N) − (1/k)] × [k/(k − 1)]}^½^ where F = the observed frequency of agreement between judges, N = the total number of judgments, and k = the number of categories [18].

Interjudge reliability scores were greater than 0.95 for category type (see Table 1) and valence (negative, neutral, positive), respectively. In light of reliability scores well above the recommended level of 0.90, six researchers (AC, BG, KH, LH, OO, JS) were trained and then individually completed six sections of the data. Thus, each of the six sections was coded by one of the six researchers after training was completed. To help assure rigor [17], random comments were selected and coded by three judges (KH, LH, JS) to make sure that consistent application of coding rules were being followed by each of the six researchers.

### 2.4. Computer-Based Text Mining

As a validity check, text mining was conducted independently by two researchers (SP, RB) who were not involved in conducting thematic analysis or content analysis. This method results in statistically driven, frequency-based clusters that are, in turn, interpreted by a content expert [19,20,21]. Raw text of written comments from survey respondents was analyzed by treating each response as a “document” and constructing a word-by-document co-occurrence matrix after stemming each word to normalize different morphological forms of the same word (e.g., convert plural nouns to singular). In addition to morphological normalization all English stopwords (e.g., “a”, “the”, “is”, etc.), symbols and numbers were removed. The word-by-document matrix was preprocessed to reduce sparseness. Hierarchical clustering was performed using Euclidian distances estimated from the word-by-document matrix and the Wald D2 method for determining clusters (number of clusters set to 10). Word clouds were generated based on frequencies obtained from the word-by-document matrix. All analyses were performed using the R statistical software package (version 3.3.2) with the “tm” text mining library, “snowball” stemming library, and “wordcloud” and “cluster” libraries used for hierarchical clustering and word cloud generation.

## 3. Results

As described previously, 2178 respondents provided written comments that were about medicines, pharmacists or health and were relevant and usable for this study. The 2178 respondents were similar to the other respondents (34,495 who did not provide comments that were usable for this study) in terms of gender (70% and 68% female, respectively), race (85% and 83% White, respectively), household income (48% and 44% $40,000 per year or less, respectively), and education (38% and 37% bachelor’s degree or more, respectively). These groups differed somewhat by age with 47% of those who wrote usable comments being age 55 and older compared with 31% in the other group being age 55 and older. Regarding health status, 35% of commenters were in fair or poor overall health compared with 30% in the other group in fair or poor overall health. Sixteen percent of those who wrote usable comments had been hospitalized within the past year compared with 13% in the other group. Regarding prescription and over-the-counter medication use, those who provided a usable comment used an average of 2.9 prescription and 1.2 over-the-counter medications daily compared with 2.8 prescription and 1.0 over-the-counter medications in the other group.

Table 2 shows that respondents to the 2015 and 2016 surveys most often commented about medication use (452 usable comments), the health care system (380 comments), and pharmacists (371 comments). In light of the objective for this study (new roles for pharmacists), we focused upon these three that were most frequently provided. Comments also were made about Pharma (349 comments), Health Care Provider (non-pharmacist)–(233 comments), Alternative Therapy (170 comments), Medication Cost (134 comments), Other (77 comments), and Caregiver (12 comments). These will be summarized with less detail and used for interpreting findings.

### 3.1. Medication Use

Table 2 shows that 66% of the comments about medication use in the 2015 and 2016 surveys had negative valences. These comments focused on lack of continuity, lack of trust, and fears associated with medication use. Representative comments about medication use include:

- It is difficult to take the best prescription for some ailments. Sometimes it is trial and error when trying to get the right prescription to work for you. That can be expensive and very frustrating to go through.
- I don’t trust drugs today because of all the side effects and long term dangers of taking them. You take one drug for something, which in turn causes something else to go wrong with your body, and you need another drug to control that problem, and yet another arises, hence another drug.
- Personally I do not like medication. It makes me feel worse than I did before I took it!
- I feel like it is extremely important for doctors to be much more careful about making sure that certain drugs are safer for the patient in question rather than carelessly prescribing whatever they think seems to work more often than not.
- It is very alarming to me that there are people who take more pills to combat side effects of a prescription they were prescribed for any given ailment. Strange.

It should be noted that 9% of the comments about medication use were neutral and 25% had positive valences. One-quarter of the comments about medications were positive and related to how medications are useful for curing and preventing disease and how medications can improve a person’s quality of life.

### 3.2. Healthcare System

Table 2 shows that 82% of the comments about the health care system in the 2015 and 2016 surveys had negative valences. These comments focused on lack of continuity, confusion, and frustration with obtaining needed services. Representative comments about the health care system include:

- Health care is much too expensive. We need to work on that!! Also insurance companies need to stop deciding what medications a person can and should use. If they trust the doctors that prescribe the drugs, then why should they be able to question the use of them?
- I believe that health care is a big, frustrating mess. I recently took myself off of all long-term medications in order to avoid it. I am now attempting to treat my issues through diet, exercise and lifestyle change.
- I wish there was a better access of information regarding where to get health care, medicine, and other such things. Specifically in the mental health field. Many times I feel like I am stuck wading through pointless information and can’t figure out how to receive proper care.
- Healthcare has become a very complex and complicated matter, very confusing for many individuals and many people have no idea how to get their questions answered. My mother used to say that once you hit 60, the medical profession really doesn’t care about you, they want to give you a prescription and make you go away.

It should be noted that 2% of the comments about the health care system were neutral and 17% had positive valences. The positive comments were about how the health care system helps make obtaining needed services accessible and affordable.

### 3.3. Pharmacists

Table 2 shows that 73% of the comments about pharmacists in the 2015 and 2016 surveys had positive valences. These comments focused on the value of the pharmacist for overcoming fears and for filling current gaps in their healthcare. Representative comments about pharmacists include:

- I love my pharmacists—they have saved the lives of me and my family members numerous times. I cannot say enough good things about my pharmacists!
- My pharmacists have gone above and beyond to learn about the experimental medications that I was on, and assisted me in trying to keep from becoming toxic in reaction with that medication and the ones I was already prescribed. Several times, I have had extreme reactions to medications. Each time, a family member was able to call my pharmacist and rush to the store to obtain a curative agent, thus preventing time in the hospital.
- My pharmacist is an important member of my healthcare team, both for me and for my mother for whom I am the caregiver. I know I can depend on the pharmacist to help me if I have concerns.
- A pharmacist once caught a drug interaction that could have caused a heart attack, I owe him my life.
- Pharmacists have to know all about diseases, drugs, side effects…they are the best source for questions about medications, both prescriptions and non-prescription…they have a big responsibility and most do an outstanding job…my hat’s off to them.
- My current pharmacist takes an active role in my health and my wife’s health. He has actively communicated with my wife’s specialist and our insurance company. He remembers who we are. If all pharmacists were like him it would be great. Some are overwhelmed with the job. He does not seem to be.
- My experience with pharmacists is that they take more time with the patient than a doctor does by making sure the patient fully understands about the drugs they’re taking and the side effects of the drugs.
- I have always relied on my pharmacists to explain my medications when I was going thru my cancer treatment. They explain everything I want and need to know in terms that I can understand. I sometimes think they know more about medicines than doctors do.

It should be noted that 5% of the comments about pharmacists were neutral and 22% had negative valences. The negative comments were about how pharmacists can be stressed and uncaring during interactions at some pharmacies.

Findings from text mining analysis validated the coding approach used for the thematic and content analyses. Text mining revealed that words such as “take and get” [22] were linked with comments about medication use. Words such as “receive and care” [23] were linked with comments about the healthcare system. Words such as “medication and like” [5,6,7,8,9,10] were associated with comments about pharmacists. These findings are consistent with—and help validate—coding decisions used and the selection of representative comments. The respondent-driven themes in the general domains of ““taking medications” [22], “receiving health care” [23], and “liking pharmacists” (pharmacist image) [5,6,7,8,9,10] are helpful for interpreting findings as well.

### 3.4. Other Categories of Comments

Table 2 shows that 96% of comments about Pharma were negative. Pharma was viewed similarly to other organizations in the health care system and were seen as creating lack of continuity, confusion, and frustration for health care consumers. If the comments about Pharma were included with other Health Care System comments, the proportion of negative comments in this category would rise to 88%.

Table 2 shows that and 97% of comments about Medication Cost were negative. The high cost of medications was given as a specific reason for making medication use difficult and was consistent with other comments about medication use. If comments about Medication Cost were included with other comments about Medication Use, the proportion of negative comments in this category would rise to 73%.

Comments about Alternative Therapy tended to be positive (87%) and identified treatment substitutes that could be tried that were not so expensive, confusing and frustrating. This reveals that individuals are seeking alternatives to the frustrations experienced in the traditional health care system and medication use processes.

Other categories of comments were mixed. Comments about Health Care Providers (non-pharmacist) were 54% negative and 42% positive. These comments highlighted both good and bad experiences with physicians, nurses, or other non-pharmacist health care providers. The “Other” category was mixed as well with 43% negative and 47% neutral. Most of the neutral comments were simply “statements of fact” without any valence connected with it. Some examples are: “I have no ailments,” “I believe in the right to choose, whether it be vaccines or medical marijuana,” and “I take three kinds of depression meds.” Finally, 12 comments were about Caregivers with 33% negative and 58% positive in valence. These highlighted both good and bad experiences with caregivers.

## 4. Discussion

Out of 2178 comments written by responders to a national on-line survey, 1203 (55%) were about medication use, the health care system, and pharmacists. Qualitative analysis of these comments showed that consumers described the lack of continuity, their lack of trust, and their fears associated with medication use. Regarding the health care system, consumers described the lack of continuity, their confusion, and their frustration with obtaining needed services. However, consumers often described pharmacists as valuable for overcoming their fears and for filling current gaps in their healthcare.

The other comments (45% of the 2178) were about Pharma, Health Care Providers (non-pharmacists), Alternative Therapy, Medication Cost, Caregivers, and Other. These comments confirmed that consumers experience lack of continuity, lack of trust, and fear associated with medication use. Also, the comments showed that consumers experience frustration with the traditional health care system and seek out alternative therapies. Some respondents reported positive experiences with other health care providers (non-pharmacists), but most reported negative experiences.

### 4.1. Pharmacists are Ideally Suited for Integrating and Coordinating Care

Since most chronic health conditions are treated and managed through medications [22,23], we propose that pharmacists are ideally situated for integrating and coordinating chronic care for health care consumers and providing continuity through transitions in care [5]. Pharmacists already have expertise in medication therapy management and could develop even greater expertise for helping health care consumers with access to, affordability of, and alternatives to medications. In their seminal work, “Through the Patient’s Eyes,” Gerteis and colleagues [24] identified seven dimensions of patient-centered care (with an eight principle—access to care—a given):

- Respect for patients’ values, preferences, and expressed needs
- **Coordination and integration of care**
- Information, communication, and education
- Physical comfort
- Emotional support and alleviation of fear and anxiety
- Involvement of family and friends
- **Transition and continuity**

The findings from our study showed that health care consumers frequently expressed a need for two of these patient-centered care principles: (1) **coordination and integration of care** and (2) **transition and continuity**. While not definitive, our analysis provided some signals that pharmacists might be viewed as a health care provider who could help meet these needs. Since consumers typically have a favorable overall perception of pharmacists [6,7,8,9], are in favor of the development of extended professional services [6,7,8], and would be willing to accept pharmacist-provided medication management services [9], we suggest that there are opportunities for new pharmacist roles in the areas of coordination and continuity of people’s health care. If new management and reimbursement systems could be created for better positioning pharmacists to provide services directly to consumers beyond their medication dispensing responsibilities [8,9,10,11,25,26,27], these opportunities could be developed into new service offerings.

Some examples of new systems that would help create and develop new product and service offerings include: (1) global population-based revenue sharing that focuses on quality and total cost of care [25]; (2) continuous medication monitoring models that apply health informatics solutions [26]; and (3) home-based models of medicines review and management [27]. Each of these examples helps integrate pharmacists into overall coordination of care with more points of interaction with consumers/patients. If these systems and new product and service offerings are put into place, the surplus of pharmacists that is predicted to come about in the United States within the next 10 years [1,2,3] would deliver the pharmacists necessary for engagement in these roles. We recommend that more research in these areas to help create new opportunities for pharmacists would be fruitful.

### 4.2. Limitations

The findings should be interpreted in light of the study’s limitations. First, the survey panels consisted of volunteers and were not random samples. However, the panels were selected to represent the U.S. adult population in terms of geographic location, gender, and age.

Second, those who took the time to write comments at the end of the survey were likely those who had an interest in the topic or had unique experiences they wanted to share. Those who made comments used in this study—compared with those who did not make usable comments—were older, on average, but otherwise were similar for other demographics (gender, race, household income, education) and health status (overall health; hospitalized in past year; daily use of prescription and over-the-counter medications).

Third, respondents were aware that the survey was sent by researchers from colleges of pharmacy. This might have biased comments to be favorable to pharmacists. We acknowledge these limitations, but the relatively large number of comments and the consistency with which certain themes emerged, provide evidence of validity for this study.

Finally, the coding applied for qualitative analysis was driven by the research team and the study objective. Qualitative inquiry using respondent-driven themes in the general domains of “taking medications” [22], “receiving health care” [23], and “liking pharmacists” (pharmacist image) [5,6,7,8,9,10] would be fruitful.

## 5. Conclusions

Consumer comments to national surveys conducted in 2015 and 2016 were generally negative regarding medication use and the health care system, but positive regarding pharmacists. These findings might serve as a signal that pharmacists could help improve coordination, integration, and continuity for peoples’ healthcare. With an increased supply of pharmacists, there is new capacity in the pharmacy profession to develop new products and services for latent markets.

## Figures and Tables

**Table 1:** Categories and Operational Definitions for Content Analysis.

Category Name	Operational Definition
1. Health Care System	Organizations and actions whose primary intent is to promote, restore or maintain health including access issues, financing, insurance, and institutions.
2. Health Care Provider (non-pharmacist)	A person (non-pharmacist) who helps in identifying or preventing or treating illness or disability as part of his or her job or licensure.
3. Pharmacist	A person (pharmacist) who is professionally qualified to prepare, dispense, and monitor medicinal drugs and works in the science or practice of pharmacy.
4. Caregiver (non-professional)	A person (non-professional) who provides direct care (as for children, elderly people, or the chronically ill). Typically a family member or relative.
5. Pharma	Pharmaceutical companies or industry including direct-to-consumer advertising and other Pharma-sponsored activities.
6. Medication Use	Utilization of medications (prescription and non-prescription) that includes their effects, safety, use patterns, and specificity to individual needs.
7. Medication Cost	Costs associated with medication use including costs to individuals, communities, and society overall.
8. Alternative Therapy	Any of various systems of healing or treating disease (as natural remedies, homeopathy, or faith healing) not included in the traditional medical curricula of the United States which focuses on medications and medical procedures.
9. Other	Any comment that does not fit into one of the other eight categories.

Development of these operational definitions was guided by the analyzed text, researcher experiences, and publicly available definitions.

**Table 2:** Content Analysis Findings.

Theme	Negative Valence	Neutral Valence	Positive Valence	Total
2015 Survey				
Medication Use	253 (66%)	38 (10%)	93 (24%)	384 (100%)
Pharma	322 (95%)	5 (2%)	10 (3%)	337 (100%)
Pharmacist	72 (22%)	20 (6%)	232 (72%)	324 (100%)
Health Care System	244 (81%)	6 (2%)	51 (17%)	301 (100%)
Health Care Provider (non-pharmacist)	70 (45%)	9 (6%)	75 (49%)	154 (100%)
Alternative Therapy	9 (6%)	12 (9%)	119 (85%)	140 (100%)
Medication Cost	100 (96%)	1 (1%)	3 (3%)	104 (100%)
Other	26 (44%)	27 (46%)	6 (10%)	59 (100%)
Caregiver	0 (0%)	0 (0%)	4 (100%)	4 (100%)
TOTAL	1096 (61%)	118 (6%)	593 (33%)	1807 (100%)
2016 Survey				
Heath Care System	66 (84%)	0 (0%)	13 (17%)	79 (100%)
Health Care Provider (non-pharmacist)	55 (70%)	0 (0%)	24 (30%)	79 (100%)
Medication Use	47 (69%)	1 (2%)	20 (29%)	68 (100%)
Pharmacist	9 (19%)	0 (0%)	38 (81%)	47 (100%)
Alternative Therapy	1 (3%)	0 (0%)	29 (97%)	30 (100%)
Medication Cost	30 (100%)	0 (0%)	0 (0%)	30 (100%)
Other	7 (39%)	9 (50%)	2 (11%)	18 (100%)
Pharma	12 (100%)	0 (0%)	0 (0%)	12 (100%)
Caregiver	4 (50%)	1 (12%)	3 (38%)	8 (100%)
TOTAL	231 (62%)	11 (3%)	129 (35%)	371 (100%)
Overall (2015 and 2016 Surveys)				
Medication Use	300 (66%)	39 (9%)	113 (25%)	452 (100%)
Health Care System	310 (82%)	6 (2%)	64 (17%)	380 (100%)
Pharmacist	81 (22%)	20 (5%)	270 (73%)	371 (100%)
Pharma	334 (96%)	5 (1%)	10 (3%)	349 (100%)
Health Care Provider (non-pharmacist)	125 (54%)	9 (4%)	99 (42%)	233 (100%)
Alternative Therapy	10 (6%)	12 (7%)	148 (87%)	170 (100%)
Medication Cost	130 (97%)	1 (1%)	3 (2%)	134 (100%)
Other	33 (43%)	36 (47%)	8 (10%)	77 (100%)
Caregiver	4 (33%)	1 (8%)	7 (58%)	12 (100%)
TOTAL	1327 (61%)	129 (6%)	722 (33%)	2178 (100%)

Totals may not sum to 100% due to rounding.

## Presentation

An earlier version of this manuscript was presented at the 2017 American Pharmacists Association Meeting, San Francisco, CA, 24 March 2017.
